# Operative Findings of over 5000 Microvascular Decompression Surgeries for Hemifacial Spasm: Our Perspective and Current Updates

**DOI:** 10.3390/life13091904

**Published:** 2023-09-13

**Authors:** Jae Sung Park, Kwan Park

**Affiliations:** 1 Department of Neurosurgery, Konyang University Hospital, 158, Gwanjeodong-ro, Seo-gu, Daejeon 35365, Republic of Korea; nsjasonpark@hotmail.com; 2 Department of Neurosurgery, Konkuk University Medical Center, 120-1, Neungdong-ro, Gwangjin-gu, Seoul 05030, Republic of Korea; 3 Department of Neurosurgery, Sungkyunkwan University School of Medicine, Seoul 06351, Republic of Korea

**Keywords:** hemifacial spasm, microvascular decompression, compressive patterns

## Abstract

Hemifacial spasm (HFS) is a hyperactive cranial neuropathy, and it has been well established that the cause of primary HFS is compression on the root exit zone (REZ) of the facial–vestibulocochlear nerve complex (CN VII-VIII) by a vessel or vessels. MVD is the only curative treatment option for HFS with a high success rate and low incidence of recurrence and complications. We categorize six classical compressive patterns on the REZ as well as five challenging types. Knowledge of these patterns may help in achieving a better surgical outcome.

## 1. Introduction

Hemifacial spasm (HFS) is a hyperactive cranial neuropathy, and it has been well established that the cause of primary HFS is compression on the root exit zone (REZ) of the facial–vestibulocochlear nerve complex (CN VII-VIII) by a vessel or vessels [1,2]. The modern understanding of its etiology has contributed to the development of microvascular decompression (MVD) which can offer a cure in a non-destructive way [3,4]. Accordingly, MVD has been accepted as the treatment of choice for medically intractable primary HFS [5]. An uncountable number of HFS patients have been benefited by MVD around the globe, but there still remains much to discover. We believe this report stands out in that it is used to describe detailed operative findings of more than 5000 MVD procedures performed by a single surgeon in a single institution. A brief overview and current updates of HFS from our experience and perspective are to be presented. 

## 2. Materials and Methods

From January 2004 to March 2020, 5026 MVDs were performed for HFS by a single surgeon in a single institution. All patients had been diagnosed with medically intractable primary HFS and underwent an MVD via a lateral retrosigmoid suboccipital approach. Preoperative evaluation included computed tomography (CT), magnetic resonance imaging (MRI) along with T2 weighted sequences, and three-dimensional time of flight MR angiography (3D TOF MRA). Neurovascular conflict is better visualized by high resolution T2 weighted images than 3D TOF MRA, especially in cases of venous compression. Pure tone audiometry along with speech audiometry were carried out before and after the MVD. 

Under general anesthesia, a lateral retrosigmoid suboccipital craniotomy was performed, followed by incision of the dura, careful dissection of the arachnoid layer, and gentle retraction of the flocculus. Upon exposure of the REZ of CN VII-VIII complex, the compressing vessel, or offending vessel, was identified. A Teflon sponge was inserted between the REZ and the offending vessel, which completed the decompression process. Throughout the surgery, electrophysiological evaluation was used to monitor the facial nerve, i.e., the disappearance of the lateral spread response (LSR), free running electromyography (EMG) and direct nerve stimulation, as well as brainstem auditory evoked potentials (BAEP), in all patients. The compression patterns were described and categorized by the surgeon (K.P.), and, according to the categorization, they were illustrated by the first author (J.P.). 

Preoperative and postoperative evaluation for symptoms were described and collected by a single nurse practitioner to minimize response bias. The data processing was carried out using commercially available software (IBM SPSS Statistics, version 24). The Chi-square test and Fisher’s exact test were employed when analyzing cross tables between compression patterns and clinical outcomes. Patient consent was not necessary because of the retrospective nature of the study, and the validity of the findings would not be affected by the absence of patient consent. Moreover, no additional risk to patient safety was expected from this study without patient consent. The first author, J.P, owns all copyright privileges regarding all illustrations. 

## 3. Results

**Figure 1:**
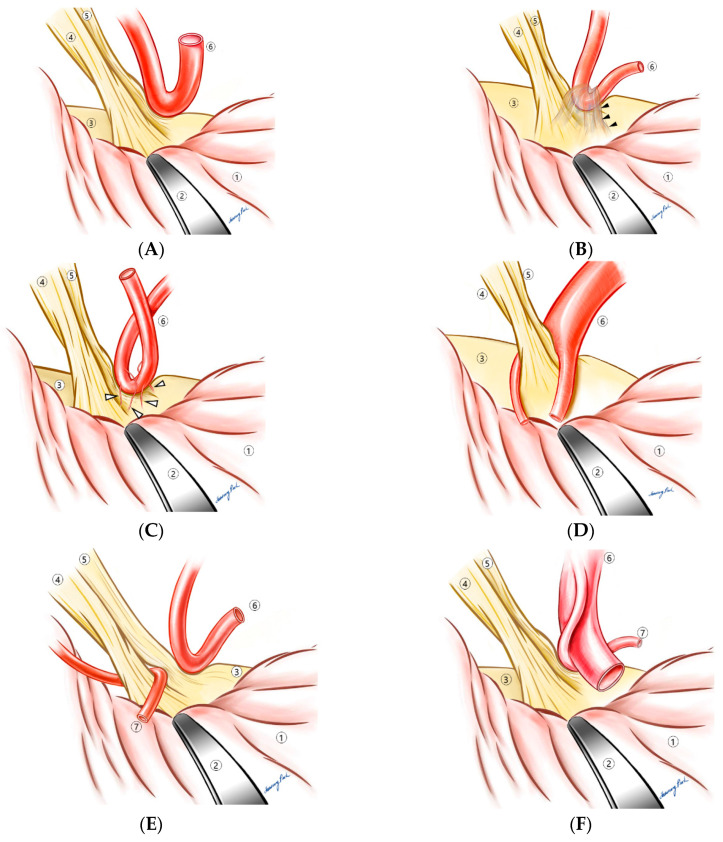
Classic compression patterns. (**A**) Loop type, (**B**) arachnoid type, black arrow heads: thickened arachnoid membrane, (**C**) perforator type, hollow arrow heads: perforating arteries, (**D**) branch type, (**E**) sandwich type, and (**F**) tandem type. ① Cerebellum, ② brain retractor, ③ brain stem, ④ vestibulocochlear nerve, ⑤ facial nerve, ⑥ primary offending vessel, and ⑦ secondary offending vessel.

**Figure 2:**
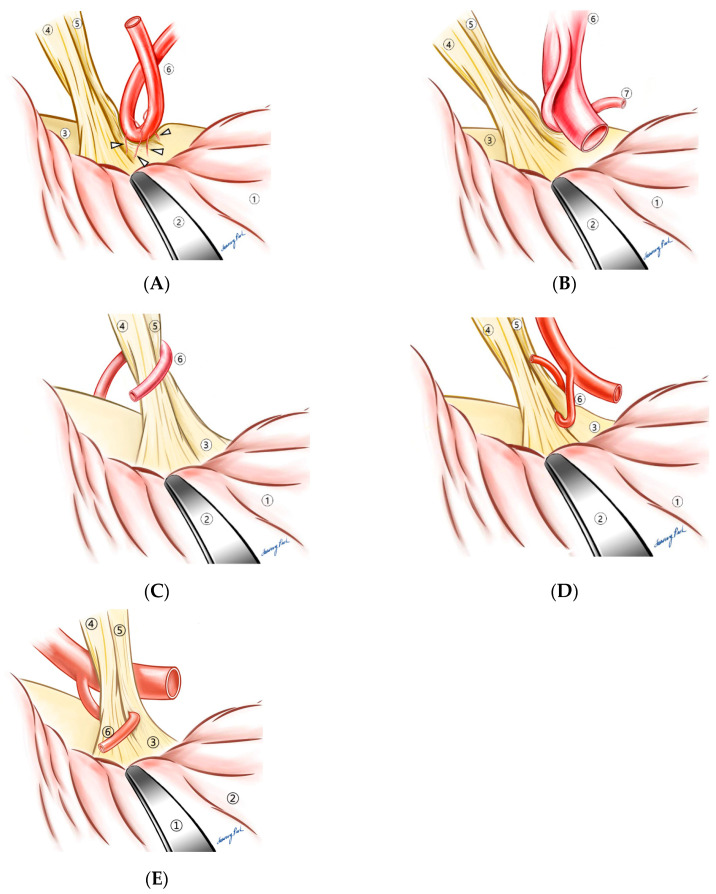
Challenging patterns. (**A**) Perforator type, hollow arrow heads: perforating arteries, (**B**) tandem type, (**C**) cisternal type, (**D**) encircling type, and (**E**) penetrating type. ① Cerebellum, ② brain retractor, ③ brain stem, ④ vestibulocochlear nerve, ⑤ facial nerve, ⑥ primary offending vessel, and ⑦ secondary offending vessel.

Over the past 16 years, operative findings were described and recorded by the surgeon (K.P.). When the REZ was inspected through a microscope, how it was compressed by a vessel or vessels was not uniform. After around 5–7 years in his career as a neurosurgeon specialized in MVD, the surgeon noticed that in the vast majority of cases, there was a contributing factor that made compression somewhat inevitable. A thickened arachnoid membrane was the first thing that inspired him to pursue this categorization process according to the contributing factors, which eventually led to the creation of the “compression patterns”. When the thickened arachnoid membrane was found around the compressing vessels, dissection of the arachnoid membrane often led to the disappearance of the LSR, which could indicate that the thickened arachnoid membrane was the cause of the compression. The surgeon hypothesized that the vessel was “forced” to compress the REZ by the thickened arachnoid membrane pushing it to the REZ (Figure 1B). Indeed, the arachnoid type, the most frequently observed one, accounts for 27.9% of all cases [6]. Under the same hypothesis, other forms of compression were described. When short and tight perforating arteries from the offending vessel were tethering the vessel to the REZ, we named it the “perforator” type (Figure 1C). This perforator type was also grouped in the challenging ones because the short and tight perforating arteries limited the working space for decompression, and they must not be injured to avoid any irreversible sequelae such as brain stem infarction or intracranial hemorrhage (Figure 2A). The compressing vessels of overall HFS, in order of frequency, consisted of the anterior inferior cerebellar artery (AICA, 51.7%), the posterior inferior cerebellar artery (PICA, 21.6%), and the vertebral artery (VA) [6]. AICA was involved in the perforating type in 84.5% of instances, which was disproportionately higher than in other type (*p* < 0.005) [6]. The branch type (Figure 1D) referred to a compression where the REZ was caught between branches of the offending artery, while the sandwich type (Figure 1E) illustrated a compression of the REZ by two independent arteries on each side. The two arteries in the sandwich type were either AICA + PICA or AICA + another branch of AICA; the VA was not involved in any sandwich types. In the tandem type, on the contrary, the VA was one of the two arteries in 61.5% of instances [6]. Figure 1F depicts the tandem type where a larger artery, most commonly the vertebral artery, compresses a smaller one that is in contact with the REZ. The tandem type was also categorized as a challenging one (Figure 2B), since Teflon pieces inserted between the REZ and the smaller offending artery might not be sufficient for a complete decompression; the smaller artery must be released from the pressure by the larger one as well. When there was no contributing factor other than the vascular loop itself, they were categorized as the “loop type” (Figure 1A). PICA was responsible for 72.7% of the loop types. 

As an addendum to the original “compression patterns”, we selected five types as “challenging patterns”, for they were found to pose an additional challenge during the process of decompression. Besides the two aforementioned types, i.e., the perforator and tandem, three unusual ones were added: cisternal, encircling, and penetrating (Figure 2). The combined number of these five types accounted for 50.4% of instances (1527 of 3028) [7]. Although these five types were named “challenging”, the clinical outcome, in terms of improvement of symptoms and postoperative complications, did not differ from that of the overall cases. The success rate of the challenging group was 88.6% whereas that of total cases was 90.1%. Likewise, the complication rate of the former was 0.71 while that of the latter was 0.89% [7]. Re-do surgeries (13 out of 1527), however, yielded a lower spasm-free rate (10 of 13, 76.9%) along with a substantially higher incidence of intraoperative BAEP change (7 of 13, 53.8%), postoperative facial palsy (6, 46.2%), and deafness (1, 7.7%) [7]. 

## 4. Discussion 

A bibliometric analysis of hemifacial spasm (HFS) in 2022 reported that the second largest number (3.26%) of all HFS-related articles in the world were published from Sungkyunkwan university in South Korea where Prof. Kwan Park initiated the hemifacial clinic and personally performed over 5000 microvascular decompressions (MVD) for HFS [8]. We hereby present a concise overview with current updates of HFS. 

### 4.1. Overview of HFS

HFS is defined as contractions on one side of the face. The clinical term, HFS, refers to involuntary facial contractions that are irregular, unilateral, and tonic or clonic. Those twitches usually start with the periorbital muscles and then they can spread to the perinasal, perioral, zygomaticus, and platysma muscles [9]. The diagnosis of HFS is primarily based on clinical history in accordance with the definition of HFS: involuntary facial contractions that are unilateral, irregular, and tonic or clonic. As an adjunctive maneuver, the “other Babinski sign”, also known as the Babinski-2 sign, may be useful. It refers to a synchronized contraction of the frontalis muscle or orbicularis oculi muscle, induced by an attempt to lift up one’s eyebrow while the eye is being closed [10]. This maneuver assists in the diagnosis of HFS with a sensitivity of 86% and a specificity of 100% [10]. Electromyography (EMG), computed tomography (CT), or magnetic resonance imaging (MRI) also can be adopted to confirm the diagnosis. Time of flight (TOF) of an MR angiography may delineate the proximity or contact of an offending vessel with the REZ. According to more recent studies where 3D MRI volumetric analysis was applied to evaluate the size of the CSF space in the posterior fossa, it appeared to be smaller in HFS patients compared to that of the control group [11]. The characteristic feature of EMG in HFS can be described as spontaneous and high-frequency synchronized firing, which may be helpful to differentiate HFS from other movement disorders, such as myokymia, blepharospasm, craniocervical dystonia (Meige syndrome), post-facial palsy synkinesia, tic disorders, myokymia, neuromyotonia, and tardive dyskinesias (TD) as well as phychogenic HFS [9]. According to an epidemiological study based on a Norwegian population, the prevalence of HFS was about 9.8 per 100,000 persons [12]. Another study from the USA reported the prevalence rate of HFS as 7.4 per 100,000 men and 14.5 per 100,000 women [13]. Data from our own institute revealed the male-to-female ratio to be 1:2.28 with an average age of 52.2 years [14]. 

The pathophysiology of HFS, as widely accepted, is explained by vascular compression on the root entry zone (REZ) of the facial nerve. When the compression of the REZ is the sole cause of HFS, it is defined as primary HFS, whereas any impairment of the facial nerve due to a pre-existing condition can constitute a secondary HFS. A modern-day concept of vascular compression syndrome that included trigeminal neuralgia, HFS, and glossopharyngeal neuralgia was introduced by McKenzie in 1936 [9]. Based on its pathophysiological background, vascular decompression for HFS was first introduced by Gardner in 1962, following which, a more modern technique with a minimal approach, i.e., MVD via retrosigmoid craniotomy, was first performed by Bremond in 1974 [9,15,16]. The current concept of the pathophysiology and surgical treatment of HFS was established and popularized by Jannetta, and it started with his article in 1975, titled “*Neurovascular cross-compression in patients with hyperactive dysfunction symptoms of the eighth cranial nerve*” [9,17]. When a vascular curvature causes the compression on the REZ, the anterior inferior cerebellar artery (AICA) is most commonly involved, followed by posterior inferior cerebellar artery (PICA), and the vertebral artery (VA). A single artery could be the sole cause of the neurovascular compression, but it is rather infrequent (4.7%) according to our previous report [9,18]. In consideration of other additional factors, a total of six compressive patterns in HFS were proposed: loop, arachnoid, perforator, branch, sandwich, and tandem types [9,18]. Regarding a more detailed mechanism of HFS in addition to the microscopic disruption of myelin in the REZ, there are two major hypotheses: the central (hyperexcitability of the facial motor nucleus) vs the peripheral (ephaptic transmission between the facial nerve bundles) hypothesis [9,19]. An increasing amount of micro-anatomical and neurophysiological research is dedicated to elucidating the precise pathway of HFS; but one hypothesis cannot explain all the phenomena without the other. 

Pharmaceutical medicine in general has failed to provide long-term improvement for HFS. Anticonvulsants or GABAergic medicines may lessen symptoms partially and temporarily, but the effectiveness of those medicines cannot be comparable to botulinum neurotoxin (BTX) injection, not to mention microvascular decompression. BTX injection is the most preferred non-surgical treatment for HFS, yielding up to 85% of symptomatic relief, and among the seven serotypes of BTX, serotypes A and B are currently commercialized [9]. Following injections, symptomatic improvement occurs in 1–3 days, and it usually reaches its peak effect in 5 days [9,20]. The duration of clinical benefit varies center to center by 3–6 months [9,21,22]. Repeated injections of BTX are unavoidable, and tolerance can naturally develop in some subjects, although a 10-year multicenter study reported that the average duration of improvement did not change from the first year of injection to the 10th year of treatment with a similar dose of BTX [9,23]. Additionally, they insisted that the adverse responses derived from BTX injections decreased throughout the 10-year course. Local complications of BTX injection include ptosis, blurred vision, and diplopia, but they are rarely permanent [9,24]. Incidence of overall adverse effects was estimated as ranging from 20 to 53%, and the most frequent one was ptosis [21,22,25]. Despite its relatively high success rate of symptomatic improvement, one cannot ignore the fact that BTX injection fundamentally requires repeated sessions, which leads to emotionally and financially non-negligible burdens on the patients [9]. 

MVD is the only curative treatment option for HFS with a high success rate and low incidence of recurrence and complications. According to a systemic review on 22 studies with 5700 patients who underwent MVD, a complete resolution was achieved in 91.1% (95% CI: 90.3–91.8%) of patients [9,26]. Recurrence occurred in 2.4% (95% CI: 1.9–2.9%) of patients, and postoperative complications included transient complications including facial palsy (9.5% [95% CI:8.8–10.3%]), hearing deficit (3.2% [95% CI: 2.7–3.7%]), and cerebrospinal fluid leak (1.4% [95% CI: 1.1–1.7%]) [9,26]. Permanent complications included hearing deficit in 2.3% (95% CI: 1.9–2.7%) and facial palsy in 0.9% (95% CI: 0.7–1.2%) of patients, the risk of stroke was 1 in 1800, and risk of death was 1 in 5500 [9,26]. 

The basic concept of MVD is well described in the literature, but the detailed techniques vary depending on institutions and surgeons. Once a lateral retro-sigmoid suboccipital craniectomy or craniotomy is performed under a general anesthesia, the dura is incised to reveal the cerebellar cortex. With or without traction of the flocculus, the root entry zone (REZ) of the facial nerve is to be observed. Upon the identification of the compressing vessels, or the offending arteries, they are separated from the seventh nerve, which then can be perpetuated by insertion of Teflon pieces. A few more additional techniques, including transposition of the vessels, snare technique, vascular sling, etc., have been proposed [9,27,28,29]. Intraoperative EMG monitoring can be beneficial for improvement of surgical outcomes. Lateral spread response (LSR) is one of the most commonly employed neurophysiologic tests for HFS since Moller and Jannetta suggested that properly performed decompression would be accompanied with the disappearance of the LSR [9,30]. However, persistence of the LSR did not necessarily indicate a poor outcome, which precludes the LSR from being a reliable predictor for the long-term prognosis of HFS after MVD [9,31]. Furthermore, to properly monitor the integrity of the eighth nerve (CN VIII) during MVD, intraoperative brain stem auditory evoked potential (BAEP) can be employed, which has been accepted by numerous institutions in decreasing the risk of hearing impairment during MVD [9]. 

Clinical courses following MVD are not identical. According to our own report, 737 (92.8%) of 807 patients who had undergone MVD for HFS became absolutely or nearly spasm free by the 2-year postoperative follow-up [9,18]. However, not everyone became asymptomatic immediately after the surgery; 140 (19.0%) of 737 patients still experienced residual spasms for more than a month, and some of them lasted more than a year [9,18]. These inhomogeneous courses of MVD for HFS may indicate that microscopic changes in the REZ, facial nerve, or facial nucleus in each patient can be diverse; some may have reversible compression without any structural changes, whereas others may have gone through microscopic changes in their facial nerve or nucleus. We believe that one cannot easily conclude the two aforementioned hypotheses of pathophysiology, i.e., hyperexcitability of the facial nucleus vs. ephaptic transmission of the facial nerve, are mutually exclusive. 

### 4.2. Compressive Patterns 

Since we introduced six compression patterns of HFS which included the loop, arachnoid, perforator, branch, sandwich, and tandem in 2008, we have received many questions concerning the significance of this categorization. As reported, one type was not necessarily associated with a better outcome compared to another, which indicated that a specific compression pattern could not determine indications for MVD. In 2020, we selected five types that can be technically challenging during MVD, but these also did not necessarily contribute to poorer results [7]. The only cases that significantly resulted in unfavorable outcomes are re-do surgeries, which underscores the importance of an accurate diagnosis, proper determination of surgical indication, thorough exploration around the REZ during MVD, and avoidance of iatrogenic compression on the REZ [7]. The significance of our categorization system can be rephrased as knowledge on these patterns may help in achieving a better surgical outcome. By being aware that there could be more than one artery causing the compression, as in the sandwich or tandem type, an incomplete decompression can be avoided. Additionally, when the vessel on the REZ is not easily movable, one must consider the possibility of the perforator type to prevent a devastating complication such as brain stem infarction or hemorrhage. Although each type did not directly impact the postoperative results, the understanding of these patterns seemed to improve the overall outcome in our institution.

A brief summary of the features and some useful tips for surgery are presented in Table 1. In the arachnoid type, the offending vessel may no longer be located on the REZ after the arachnoid dissection, which can coincide with the disappearance of the LSR. When the preoperative MRI delineates the VA near the REZ, the tandem type should be the first one to rule out. If the tandem type is confirmed during the surgery, one should be reminded of the fact that the simple insertion of Teflon pieces between the REZ and the smaller artery may not guarantee a satisfactory outcome, because the larger artery, most likely the VA, would probably continue to derive throbbing forces to the smaller one. The larger artery must be detached from the smaller one, and furthermore, it would be ideal if the larger one could be transposed so that the trajectory of the throbbing force can be re-directed. The loop type is the least challenging since the approach to the REZ is not hindered by any obstacles and there is enough working space for decompression. The involvement of PICA is most frequent in this type. The perforator type is one of the challenging ones. Owing to the tight perforators, manipulation of the compressing artery can be highly difficult and sometimes potentially dangerous. Any disruption to one or more of the perforators can result in permanent impairment to the brain stem. When the REZ is caught between the branches, the main trunk must be moved off the REZ, so that the inserted Teflon does not cause an iatrogenic compression. The sandwich type can be overlooked if the medial side of the REZ is not thoroughly inspected. After one compression on the dorsal side of the REZ is successfully decompressed, the medial side also should be carefully observed. 

The arachnoid type was most frequently observed (27.9%), followed by the tandem (24.6%) and the perforator (22.0%) types based on our own research in 2008 [6]. The addendum classification was published in 2021, where 1527 (50.4%) of 3027 MVD for HFS cases were selected as such [7]. Among the challenging types, the tandem (40.2%) and the perforator (31.1%) accounted for the majority, and the remaining three types included cisternal, encircling, and penetrating ones in order of frequency; the penetrating type was the most extreme one (4 out of 3027, 0.13%). In the cisternal type, it is sometimes difficult to find the compression, because the usual compression site, i.e., the REZ, appears to be compression-free. Exploration towards the cisternal portion of the facial nerve often demands further retraction of the cerebellum, but special attention must be paid not to retract it excessively. BAEP monitoring is mandatory. When the encircling artery is found around the REZ, the decompression process should be carried out from the medial to the lateral side of the REZ, because the insertion of Teflon pieces on the lateral side may hinder the medial side from being properly maneuvered. The penetrating type is the most rare and probably the most challenging of all. These challenging types did not necessarily lead to poorer outcomes, whereas revision cases resulted in a significantly lower spasm-free rate (10 of 13, 76.9%) along with a substantially higher incidence of intraoperative BAEP change (7 of 13, 53.8%), postoperative facial palsy (6, 46.2%), and deafness (1, 7.7%) [7]. 

Veins were found to be responsible for HFS in 6 (1.1%) out of 528 HFS patients, according to our previous study; 2 of them were solely caused by venous compression, whereas the remaining 4 cases were due to a combination of arterial and venous compression [32]. We did not cauterize the vein as it may result in venous infarction or brain stem injury. After careful manipulation of the vein, the REZ was decompressed using a small piece of Teflon. Although we do not have statistically significant data, HFS due to venous compression appeared to be associated with rather unfavorable outcomes because it was not always possible to detach the vein from the REZ and manipulation of the facial nerve was sometimes inevitable. 

The importance of a thorough 360° inspection around the REZ cannot be overemphasized. Even after one offending artery is successfully detached and insulated, the medial and cisternal sides of the REZ should also be free from any compression. The LSR tends to disappear when the REZ is no longer compressed, but it is questionable if the disappearance of the LSR can be a predictor of the long-term prognosis [18,24]. If the LSR still exists after a decompression process, it could indicate either incomplete decompression with a secondary cause of compression untreated, or complete decompression with lingering hyperexcitability of the facial nucleus or ephaptic transmission between nerve fibers [2,14,33]. Since it is not possible to discriminate the latter from the former during a surgery, a hypothetical secondary cause should always be ruled out to achieve a long-term cure. 

### 4.3. Brief Summary of Current Updates

A recent randomized clinical trial (RCT) on BTX injections for HFS revealed that bilateral injection of BTX decreased facial asymmetry more than ipsilateral injection did [34]. In a related study, MR tractography findings were evaluated in HFS patients following injection of BTX, where apparent diffusion coefficient (ADC) and fractional anisotropy (FA) values in the contralateral motor cortex were found to be close to those of the pathological side [35]. The authors suggested that this result might indicate an impact of peripherally injected BTX on the central nervous system [35]. Another double blinded, RCT demonstrated that pretarsal injection of BTX was more efficient than preseptal injection in terms of better symptom control and longer duration of efficacy [36].

Surgical techniques have evolved over time as well. Since the concept of MVD initiated by Jannetta et al. has been employed for HFS, trigeminal neuralgia, and glossopharyngeal neuralgia, newer techniques have emerged. The “transposition technique” differs from the simple insertion of Teflon pieces as it aims to alter the location and trajectory of the offending vessel so that the vessel can no longer convey the pulsating force to the REZ [37]. This technique can be roughly rephrased as “off the REZ”, since the REZ is free not only from the offending vessel but also from any iatrogenic Teflon pieces [37]. MVD using this transposition technique, with or without the help of a fibrin coated sling, demonstrated a higher success and lower recurrence rate [37]. As we emphasized, the re-do surgeries resulted in significantly poorer outcomes [7]. During a revision MVD, previously inserted Teflon pieces were often found near the REZ, and they were thought to have accounted for the residual or recurrent symptoms. Iatrogenic compression must be avoided at all costs, and we believe that this “off the REZ” policy may be the key to prevent a recurrence, and accordingly, a re-do intervention. 

Endoscope-assisted MVD and fully endoscopic surgery have gained increasing popularity. A meta-analysis comparing the traditional and endoscopic MVD, with a total of 12 studies and 1122 patients, reported that the endoscopic MVD yielded a higher success rate (97% vs. 89%), lower recurrence rate (5.7% vs. 0.3%), as well as a lower complication rate (12% vs. 27%) than the microscopic MVD did [38]. Another study in 2019 also insisted that a fully endoscopic MVD is both safe and feasible in the treatment of HFS since it can provide a better visualization of the neurovascular conflict, despite its original shortcomings, e.g., being prone to blood soiling, lacking 3D information, or having a longer learning curve [39]. 

The disappearance of the LSR is still a useful parameter during MVD, either traditional or endoscopic, as the majority of researchers concur. A meta-analysis on intraoperative monitoring of the LSR reported that an intraoperative disappearance of the LSR could predict a favorable clinical outcome with a high specificity of 90% at discharge and after 1 year, whereas the sensitivity was only 40% at discharge and after 1 year [40]. We believe this lower sensitivity of the LSR might be derived from the hyperexcitability of the facial motor nucleus in HFS, which could be sustained even after a successful decompression. A neurophysiological study using a novel parameter in the future may distinguish the hyperexcitability of the facial motor nucleus from the ephaptic transmission. 

## 5. Conclusions 

We believe the categorization of compressing patterns on the REZ of HFS patients as well as insightful technical tips in accordance with each individual pattern, may contribute to a safer and more efficient MVD for HFS. The golden rules of successful MVD for HFS are accurate diagnosis, proper indication for surgery, thorough and careful exploration around the REZ, and avoidance of iatrogenic compression on the REZ. 

## Figures and Tables

**Table 1:** Features and surgical tips for each type.

	Distinctive Feature	Tips for Operation
Classic compression patterns		
* Loop type*	Compression most commonly by PICA ^¶^	Technically least challenging
* Arachnoid type*	Thickened arachnoid membrane	1. Careful and thorough dissection of the arachnoid membrane is the key ^¶^ 2. LSR * may disappear upon the release of the arachnoid membrane
* Perforator type*	Tight perforators	Do not attempt to move the vessel off the REZ forcefully or excessively
* Branch type*	Caught between branches	Make sure the main trunk is moved off the REZ
* Sandwich type*	Two independent arteries from each side	After a successful decompression, always consider another possible source of compression
* Tandem type*	Larger vessel (most commonly VA ^β^) compressing a smaller one	Trajectory of the larger vessel must be altered to accomplish a full decompression
Challenging patterns		
* Perforator type*	Written above	Written above
* Tandem type*	Written above	Written above
* Cisternal type*	Compression on a cisternal portion of the facial nerve, instead of the REZ ^δ^	Be careful not to excessively retract the cerebellum
* Encircling type*	More than 270° contact with the nerve	Decompression process should be carried out from medial to lateral side of the REZ
* Penetrating type*	Most rare (0.13%)	Extra care must be taken not to injure the facial nerve

^¶^: Posterior inferior cerebellar artery, ^β^: vertebral artery, ^δ^: root exit zone, *: lateral spread response.

## Data Availability

Data available on request due to restrictions eg privacy or ethical.
